# Enhanced antiproliferative and apoptotic response to combined treatment of γ-tocotrienol with erlotinib or gefitinib in mammary tumor cells

**DOI:** 10.1186/1471-2407-10-84

**Published:** 2010-03-08

**Authors:** Sunitha V Bachawal, Vikram B Wali, Paul W Sylvester

**Affiliations:** 1 College of Pharmacy, University of Louisiana at Monroe, 700 University Ave, Monroe, Louisiana 71209, USA

## Abstract

**Background:**

Aberrant ErbB receptor signaling is associated with various types of malignancies. γ-Tocotrienol is a member of the vitamin E family of compounds that displays potent anticancer activity that is associated with suppression in ErbB receptor phosphorylation and mitogenic signaling. Erlotinib and gefitinib are tyrosine kinase inhibitors that block ErbB1 receptor activation, whereas trastuzumab is a monoclonal antibody that has been designed to specifically inhibit ErbB2 receptor activation. However, the clinical effectiveness of these agents have been disappointing because of cooperation between different ErbB family members that can rescue cancer cells from agents directed against a single ErbB receptor subtype. It was hypothesized that targeting multiple ErbB receptor subtypes with combined treatment of γ-tocotrienol and ErbB receptor inhibitors would provide greater anticancer effects than monotherapy targeting only a single ErbB receptor subtype.

**Methods:**

Highly malignant mouse +SA mammary epithelial cells were maintained in culture on serum-free defined media containing 10 ng/ml EGF as a mitogen. Cell viability wase determined by MTT assay, whereas Western blot and immunofluorescent staining was used to determine treatment effects on ErbB receptor subtype level and activation. Treatment-induced apoptosis was determined using annexin V staining and Western blot analysis of cleaved caspase-3 and PARP levels.

**Results:**

Treatment with 3.5 μM γ-tocotrienol, 0.5 μM erlotinib or 1.0 μM gefitinib alone, significantly inhibited +SA tumor cell growth. Combined treatment with subeffective doses of erlotinib (0.25 μM) or gefitinib (0.5 μM) with subeffective doses of γ-tocotrienol (0.5-3.0 μM) significantly inhibited the growth and induced apoptosis in a dose-responsive manner. Trastuzumab treatment alone or in combination had no effect on +SA cell growth and viability. Combined treatment of γ-tocotrienol with erlotinib or gefitinib also cause a large decrease in ErbB3, ErbB4, and to a lesser extent ErbB2 receptor levels, and EGF-dependent ErbB2-4 tyrosine phosphorylation (activation), but had no effect on ErbB1 receptor levels or activation.

**Conclusion:**

Combination treatment of γ-tocotrienol with specific ErbB receptor inhibitors is more effective in reducing mammary tumor cell growth and viability than high dose monotherapy, suggesting that targeting multiple ErbB receptors with combination therapy may significantly improve the therapeutic response in breast cancer patients.

## Background

γ-Tocotrienol is a member of the vitamin E family of compounds that displays potent anticancer activity at treatment doses that have little or no effect on normal cell function or viability [1-4]. Studies have shown that the growth inhibitory effects of γ-tocotrienol result from a suppression in EGF-dependent ErbB3 receptor activation and subsequent reduction in phosphatidylinositol 3-kinase (PI3K)/Akt mitogenic signaling [5]. EGF-receptors belong to the HER (human) or ErbB (mouse) family of receptor tyrosine kinases and include four members classified as ErbB1-4. Various EGF-like receptor ligands bind to and activate ErbB1, ErbB3 and ErbB4 receptors, leading to the formation of receptor homo- and heterodimers [6-8]. Receptor dimerization allows for transphosphorylation of specific tyrosine residues located on the intracellular domain which are required for substrate interaction and downstream activation of mitogenic signaling pathways [6,9]. Although the ErbB2 receptors lack a ligand binding site and the ErbB3 receptor has no tyrosine kinase activity, these receptors can initiate signal transduction by undergoing homo- or heterodimerization [10,11] and are particularly potent in activating PI3K/Akt and Ras/mitogen activated protein kinase (MAPK) mitogenic signaling pathways, and elevated PI3K/Akt and MAPK activity is associated with advanced tumor progression and poor prognosis in breast cancer patients [6,9,12,13].

ErbB receptors are expressed in a wide range of cell types and play an important role in normal cell proliferation and survival. However, aberrant ErbB receptor signaling is often associated with the development of various types of human malignancies. Two important strategies have been developed to target ErbB receptors in cancer. One strategy uses monoclonal antibodies directed towards the extracellular domain of ErbB receptors, while the other strategy uses small molecule tyrosine kinase inhibitors (TKIs) that compete with ATP for binding to the kinase domain of the receptor. Erlotinib and gefitinib are TKIs that compete with ATP for binding to the intracellular catalytic domain of ErbB1 receptor, and reversibly inhibit the phosphorylation and signal transduction events associated with ErbB1 receptor activation [14]. Trastuzumab is a monoclonal antibody that has been designed to specifically inhibit the activation of ErbB2 receptor [15].

Unfortunately, the clinical usefulness of agents that target only a single ErbB receptor subtype has been limited due to cooperation between the different ErbB family members that leads to the formation of heterodimers that are able to circumvent and rescue cancer cells from the inhibitory effects of these agents [9,16-18]. However, the therapeutic efficacy of ErbB receptor inhibitors can be improved by interfering with the cooperation among different ErbB receptor family members [19,20]. Since previous studies showed that the antiproliferative effects of γ-tocotrienol are associated with suppression in ErbB3 receptor activation and mitogenic signaling, studies were conducted targeting multiple ErbB receptors with combined treatment of γ-tocotrienol with ErbB receptor inhibitors to determine if combined treatment provided a greater anticancer response as compared to monotherapy that targets only a single ErbB receptor subtype.

## Methods

### Reagents and Antibodies

All reagents were purchased from Sigma Chemical Co. (St Louis, MO.), unless otherwise stated. Antibodies for ErbB1 (# 2232), phospho-ErbB1 (Tyr 1173) (# 4407), ErbB2 (# 2242), phospho-ErbB2 (Tyr 877) (# 2241), ErbB3 (# 4754), phospho-ErbB3 (Tyr 1289) (# 4791), phospho-ErbB4 (Tyr 1284) (# 4757), caspase-3 (# 9662), cleaved caspase-3 (# 9661), PARP (# 9542), and cleaved PARP (# 9544), for use in Western blot analysis were purchased from Cell Signaling Technology (Beverly, MA). ErbB4 receptor antibody (# RB-284-P0) was purchased from Neomarkers (Fremont, CA). Antibodies for ErbB1 (sc-03), ErbB2 (sc-284), ErbB3 (sc-285), ErbB4 (sc-883), phospho-ErbB2 (sc-12352), phospho-ErbB4 (sc-33040) and their respective blocking peptides were purchased from Santa Cruz Biotechnology Inc. (Santa Cruz, CA) for immunofluorescence studies. Goat anti-rabbit (NEF812001EA) and goat anti-mouse (NEF822001EA) secondary antibodies were obtained from PerkinElmer Biosciences (Boston, MA). Polyclonal anti-tubulin antibody (CP06) was purchased from Calbiochem (San Diego, CA). Rhodamine red conjugated goat anti-rabbit secondary antibody (R6394) was purchased from Molecular Probes (Invitrogen Corporation, Carlsbad, CA).

### Cell Line and Culture Conditions

Experiments conducted in the present study represent a logical continuation of previous studies that have extensively characterized the antiproliferative and apoptotic effects of γ-tocotrienol in the highly malignant +SA mammary epithelial cell line [21]. The neoplastic +SA cell line was derived from a mammary adenocarcinoma that developed spontaneously in a BALB/c female mouse. The +SA cell line is characterized as being highly malignant, estrogen-independent, and displays anchorage-independent growth when cultured in soft agarose gels [22,23]. Cell culture and experimental procedures have been previously described in detail [2,3,24]. Briefly, +SA cells were maintained in serum-free defined DMEM/F12 media (supplemented with 5 mg/ml BSA, 10 μg/ml insulin, 10 μg/ml transferrin, 100 U/ml soybean trypsin inhibitor, 1 μg/ml d-α-tocopherol, 100 U/ml penicillin, 0.1 mg/ml streptomycin, and 10 ng/ml EGF) at 37°C in an environment of 95% air and 5% CO_2 _in humidified incubator. For plating cells at the start of each experiment, cells were isolated with trypsin, pelleted by centrifugation, and then resuspended in serum-free defined medium, and counted by hemocytometer. Cells were plated at a density of 5 × 10^4 ^cells/well in 24-well culture plates (growth studies) or 2 × 10^5 ^cells/well in 6-well culture plates (immunofluorescence studies) or 1 × 10^6 ^cells/100-mm culture plates (all other studies) and allowed to attach in serum-free defined media for 24 h.

### Experimental Treatments

γ-Tocotrienol was generously provided by First Tech International Ltd. (Hong Kong), erlotinib and trastuzumab were provided as a gift by Genentech (San Francisco, CA), and gefitinib was kindly provided by AstraZeneca (Cheshire, UK). The highly lipophilic γ-tocotrienol was suspended in a solution of sterile 10% BSA as described previously [2,24]. Briefly, an appropriate amount of γ-tocotrienol was dissolved in 100 μl of 100% ethanol, and then added to a small volume of sterile 10% BSA in water and incubated overnight at 37°C. This γ-tocotrienol/BSA solution was then used to prepare various concentrations of treatment media. All control and treatment media had a final concentration of 5 mg/ml BSA, and ethanol was added to all treatment media such that the final concentration was the same in all treatment groups with in a given experiment and was always less than 0.1%. Stock solutions of erlotinib and gefitinib were prepared in DMSO and added to the culture medium so that the concentration of DMSO never exceeds 0.1%. Trastuzumab was dissolved in sterile water.

### Measurement of Viable Cell Number

+SA cell number was measured by 3-(4, 5-dimethylthiazol-2yl)-2,5-diphenyl tetrazolium bromide (MTT) colorimetric assay as described previously [2,5,24-26]. After the end of the treatment period, control and treatment media were replaced with fresh serum-free control defined media containing 0.41 mg/ml MTT and returned to the incubator for a period of 4 h. After incubation, the media was removed and MTT crystals were dissolved in 1 ml of isopropanol. The optical density of each sample was measured at 570 nm on a microplate reader (SpectraCount, Packard BioScience Company, Meriden, CT) zeroed against a blank prepared from cell free medium. The number of cells per well was calculated against a standard curve prepared by plating known concentrations of cells, as determined by hemocytometer, in triplicate at the start of each experiment.

### Western Blot Analysis

After the end of the treatment period, cells in the various treatment groups were isolated with trypsin, and whole cell lysates were prepared and dissolved in Laemmli buffer as previously described, [5,24,26] and protein concentration in each sample was determined by using BioRad protein assay kit (BioRad, Hercules, CA). Equal amounts of protein from each sample (25-40 μg/lane) in a given experiment was loaded on SDS-polyacrylamide minigels and electrophoresed through 5%-15% resolving gel. Each gel was then equilibrated in transfer buffer and transblotted for 12-16 h at 4°C at 30 mV to PVDF membranes (Dupont, Boston, MA) according to method of Towbin [27]. The membranes were blocked with 2% BSA in 10 mM Tris HCl containing 50 mM NaCl and 0.1% Tween 20 pH 7.4 (TBST) and incubated with specific primary antibodies against total and phosphorylated ErbB1-4 receptors, caspase-3, cleaved caspase-3, PARP, and cleaved PARP in TBST containing 2% BSA for 2 h. Membranes were then rinsed 5 times with TBST for 25 min followed by incubation with horseradish peroxide conjugated goat anti-rabbit or goat anti-mouse antibody in TBST containing 2% BSA for 1 h. Blots were then rinsed 5 times with TBST buffer and the protein bands were then visualized by chemiluminescence according to the manufacturer's instructions (Pierce, Rockford, IL). The visualization of α-tubulin was used to confirm that there was equal sample loading in each lane.

### Measurement of Annexin V Staining

Analysis of annexin V binding in each treatment group was determined using Annexin V-FITC Apoptosis Detection Kit I (BD PharMingen, San Diego, CA.) as described previously [28]. Briefly, cells in each treatment group were harvested and resuspended in annexin V binding buffer at a concentration of 1 × 10^6 ^cells/ml. Later, 5 μl of annexin V and 5 μl of propidium iodide was added to 100 μl of the suspension in triplicate and incubated in dark for 15 min at room temperature. Samples were then diluted to 500 μl with binding buffer and analyzed by flow cytometer (FACS-Calibur, BD Biosciences, San Jose, CA). Data was processed using CellQuest software (BD Biosciences).

### Immunofluorescent Staining

Neoplastic +SA cells in various treatment groups were rinsed twice with 0.05 M Tris buffered saline (TBS) pH 7.6, fixed with methanol previously cooled to -20°C and blocked with 2% goat serum in TBS for 30 min. Cells were then incubated with specific primary antibodies against ErbB1-4, phospho-ErbB2 or phospho-ErbB4 receptors overnight at 4°C in 2% goat serum in TBS. After washing the cells 5 times with TBS, cells were incubated in dark with rhodamine conjugated goat anti-rabbit antibody in 2% goat serum in TBS. After 5 final washings, cells were embedded in Vectashield Mounting Medium (Vector Laboratories Inc., Burlingame, CA). Staining was examined by Nikon ECLIPSE TE2000-U microscope (Nikon Instruments Inc., Melville, NY) coupled with CoolSNAP *cf *CCD camera (Roper Scientific Inc, Photometrics, Tucson, AZ). Digital images were captured using Metamorph software (Universal Imaging Corporation). Gray scale images thus obtained were pseudo-colored and the fluorescence observed for each ErbB receptor was assigned a different color. ErbB1, 2, 3 and 4 receptors were assigned blue, green, red and yellow colors respectively. The specificity of each antibody used in the immunofluorescent staining was verified using a blocking peptide that corresponded to the epitope recognized by the primary antibody.

### Statistical Analysis

Differences among various treatment groups in growth studies were determined by analysis of variance followed by Dunnett's *t *test. Differences were considered statistically significant at a value of *P *< 0.05. The level of interaction between the ErbB receptor inhibitors and γ-tocotrienol was determined by combination index (CI) analysis as described previously [26]. The combination index (CI) is a quantitative representation of pharmacological interaction between two drugs. The CI value <1, 1 and >1 indicate synergistic, additive and antagonistic effects respectively. The CI values are calculated as follows

*X *and *T *stand for the concentrations of ErbB inhibitor and γ-tocotrienol respectively, that induce 50% cell growth inhibition; *Xc *and *Tc *are the concentrations of ErbB inhibitor and γ-tocotrienol that induce 50% cell growth inhibition when used in combination as determined by nonlinear regression curve fit analysis using GraphPad Prism 5 software.

## Results

### Antiproliferative effects of γ-tocotrienol, erlotinib, gefitinib and trastuzumab

**Figure 1:**
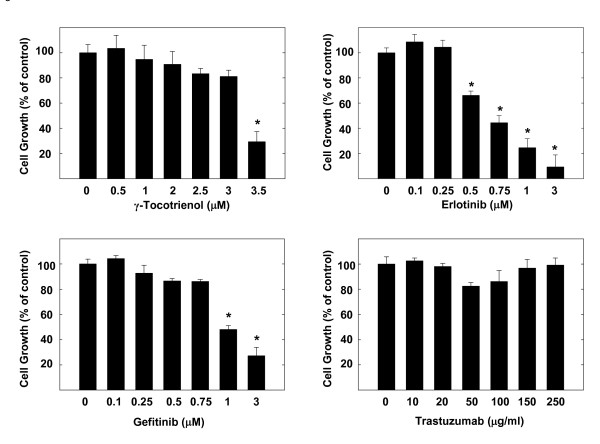
**Antiproliferative effects of γ-tocotrienol, erlotinib, gefitinib, and trastuzumab on neoplastic +SA mammary epithelial cell growth**. Cells were plated at a density of 5 × 10^4 ^cells per well (6 replicates/group) in 24-well plates and exposed to their respective treatments for a 4-day period. Viable cell number was determined by MTT colorimetric assay and indicated as percentage of control. Each bar represents the mean ± SEM in each treatment group and is representative of three independent experiments. *P < 0.05 as compared to the vehicle treated controls.

Treatment effects on the growth of highly malignant +SA mouse mammary epithelial cells over a 4-day culture period are shown in Figure 1. Treatment with 3.5 μM γ-tocotrienol, 0.5-3 μM erlotinib, or 1-3 μM gefitinib significantly inhibited EGF-dependent +SA cell growth as compared to the vehicle treated control group. However, treatment with 0-250 μg/ml trastuzumab had no effect on +SA cell growth (Figure 1).

### Antiproliferative effects of combined γ-tocotrienol treatment with erlotinib, gefitinib or trastuzumab

**Figure 2:**
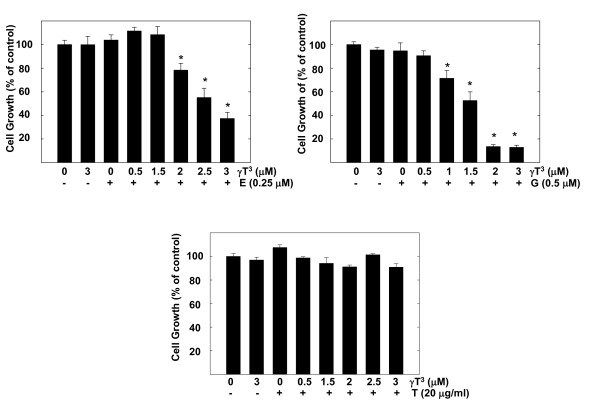
**Effects of various subeffective doses of γ-tocotrienol (γT^3^) when given alone or in combination with subeffective dose of erlotinib (E), gefitinib (G), or trastuzumab (T) on neoplastic +SA mammary epithelial cell growth**. Cells were plated at a density of 5 × 10^4 ^cells per well (6 replicates/group) in 24-well plates and exposed to respective control and treatment serum-free defined media for a 4-day period. Viable cell number was determined by MTT colorimetric assay and indicated as percentage of control. Each bar represents the mean ± SEM in each treatment group and is representative of three independent experiments. *P < 0.05 as compared to the vehicle treated controls.

**Figure 3:**
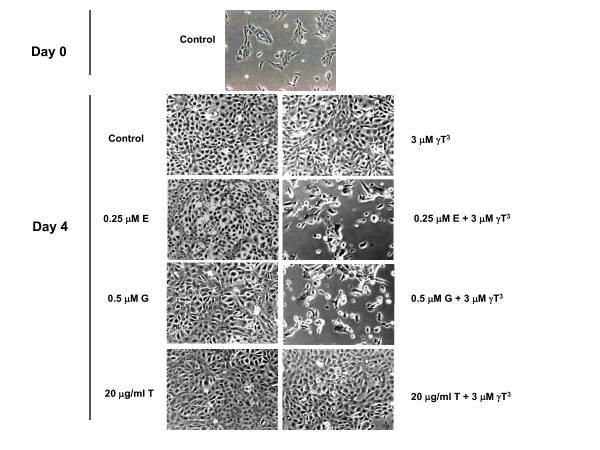
**Photomicrographs of neoplastic +SA mammary epithelial cells in culture treated with a subeffective dose of γ-tocotrienol (γT^3^) alone or in combination with a subeffective dose of erlotinib (E), gefitinib (G), or trastuzumab (T)**. Cells were plated at a density of 5 × 10^4 ^cells (Day 0) per well (6 replicates/group) in 24-well plates and exposed to respective control and treatment serum-free defined media for a 4-day period. Magnification is 100×.

In combination studies, a range of subeffective doses of γ-tocotrienol (0.5-3 μM) were combined with subeffective doses of erlotinib (0.25 μM), gefitinib (0.5 μM) or trastuzumab (20 μg/ml), to determine treatment effect on +SA cell growth over a 4-day culture period. Figure 2 shows that treatment with 3 μM γ-tocotrienol, 0.25 μM erlotinib, 0.5 μM gefitinib or 20 μg/ml trastuzumab had no significant effect on +SA cell growth as compared to vehicle-treated controls (Figure 2). However, combined treatment with similar doses of erlotinib or gefitinib with a range of subeffective doses of γ-tocotrienol (0.5-3 μM) induced a significant reduction in the EGF-dependent +SA cell growth (Figure 2). In contrast, combined treatment of 20 μg/ml trastuzumab with 0.5-3 μM γ-tocotrienol had no effect on +SA cell growth (Figure 2). The CI values for the combination of erlotinib with γ-tocotrienol (0.96) and gefitinib with γ-tocotrienol (0.91) is <1 and indicate a synergistic interaction of these drug combinations. It was not possible to calculate the CI value for the combination of trastuzumab with γ-tocotrienol because the dose at with the trastuzumab induced 50% growth inhibition could not be determined using the doses tested in the current study. Figure 3 shows photomicrographs of +SA cells in the various treatment groups described in Figure 2. Treatment of erlotinib or gefitinib in combination with γ-tocotrienol resulted in a suppression in +SA cell growth and a slight change in cellular morphology from a flat to a more elongated and round in shape, indicating cellular stress and the initiation of apoptosis (Figure 3).

### Apoptotic effects of Combined γ-Tocotrienol Treatment with Erlotinib, Gefitinib or Trastuzumab

**Figure 4:**
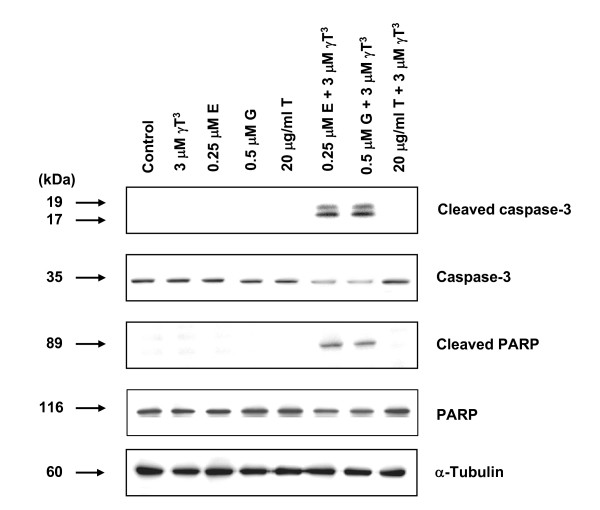
**Western blot analysis of γ-tocotrienol (γ T^3^), erlotinib (E), gefitinib (G), or trastuzumab (T) treatment effects given alone and in combination on the relative levels of cleaved caspase-3, caspase-3, cleaved PARP and PARP in neoplastic +SA mammary epithelial cells after a 4-day incubation period**. +SA mammary tumor cells were plated at a density of 1 × 10^6 ^cells/100 mm culture dish and incubated with control or treatment defined media over a period of 4-days. Afterwards, whole cell lysates (30 μg) from different treatment groups were prepared for subsequent separation by PAGE followed by Western blot analysis. Each Western blot is a representative image of data obtained for experiments that were repeated at least three times.

The effects of combined γ-tocotrienol treatment with erlotinib, gefitinib or trastuzumab on cleaved caspase-3 (activated) and cleaved PARP levels (markers for apoptosis) in +SA cells following a 4-day culture period are shown in Figure 4. Western blot analysis showed that combined γ-tocotrienol treatment with erlotinib or gefitinib, but not trastuzumab, increased the levels of cleaved caspase-3 and cleaved PARP.

**Figure 5:**
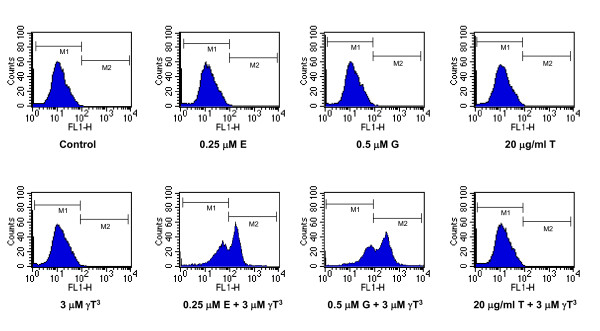
**Effects of combined γ-tocotrienol (γT^3^), erlotinib (E), gefitinib (G), or trastuzumab (T) treatment on positive annexin V staining in +SA cells after a 4-day incubation period**. +SA mammary tumor cells were plated at a density of 1 × 10^6 ^cells/100-mm culture dish and incubated with control or treatment media over a 4-day period. Afterwards, cells were isolated with trypsin, and 1 × 10^6 ^cells/group in triplicate were stained with annexin V, and then analyzed by flow cytometry. Viable cells display very low annexin V staining and appear in the marker 1 (M1) region, whereas cells undergoing apoptosis show very high annexin V staining and appear in the marker 2 (M2) region of the histogram.

Figure 5 shows the effect of the various treatments on annexin V staining in +SA cells following a 4-day culture period. Viable cells in the control group displayed very low annexin V staining and they appear in the M1 region of the histogram (Figure 5). When the cells undergo apoptosis, there will be an increase in the cells staining positive to annexin V and results in the shift of the histogram from M1 to M2 region. In control, 0.25 μM erlotinib, 0.5 μM gefitinib, 20 μg/ml trastuzumab, 3 μM γ-tocotrienol and the combination of 20 μg/ml trastuzumab and 3 μM γ-tocotrienol treated groups more than 96% of the cells appear in the M1 region where as the percentage of cell appearing in the M1, M2 regions in combined γ-tocotrienol and erlotinib or gefitinib is 48.03%, 51.48% and 36%, 63.24% respectively as determined by the CellQuest software. These results indicate that combined γ-tocotrienol treatment with erlotinib or gefitinib, but not trastuzumab, increased the percentage of cells appearing in M2 region (Figure 5)

### EGF-induced ErbB Receptor Phosphorylation

**Figure 6:**
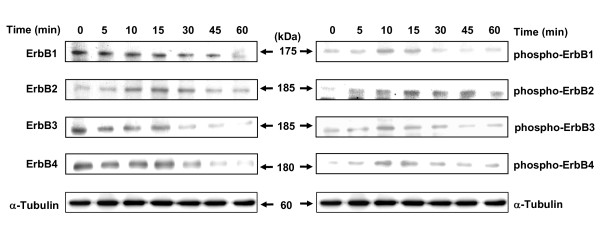
**Total ErbB and phosphorylated ErbB (activation) receptor levels in neoplastic +SA mammary epithelial cells**. +SA mammary tumor cells were initially plated a density of 1 × 10^6 ^cells/100 mm culture dish. Afterwards, cells were incubated overnight in mitogen-free media. Cells in all treatment groups were then stimulated with 100 ng/ml EGF for 0-60 min. Following treatment exposure, whole cell lysates were prepared and then subjected to PAGE followed by Western blot analysis for total and phosphorylated (active) ErbB receptor levels. Each Western blot is a representative image of data obtained for experiments that were repeated at least three times.

Western blot analysis showed that following exposure to EGF, there was a gradual decrease in the total levels of ErbB1, ErbB3 and ErbB4 receptors over a 60 min time period, while ErbB2 receptor levels showed a transient increase and then decrease in levels following EGF exposure (Figure 6). EGF exposure resulted in an increase in tyrosine phosphorylation (activation) in all ErbB receptor family members (ErbB1-4), with maximal phosphorylation observed 10 min after EGF exposure followed by a subsequent decrease in ErbB1-4 receptor tyrosine phosphorylation, reflecting the internalization and degradation of ErbB1-4 heterodimer receptor complexes following EGF-induced activation (Figure 6).

**Figure 7:**
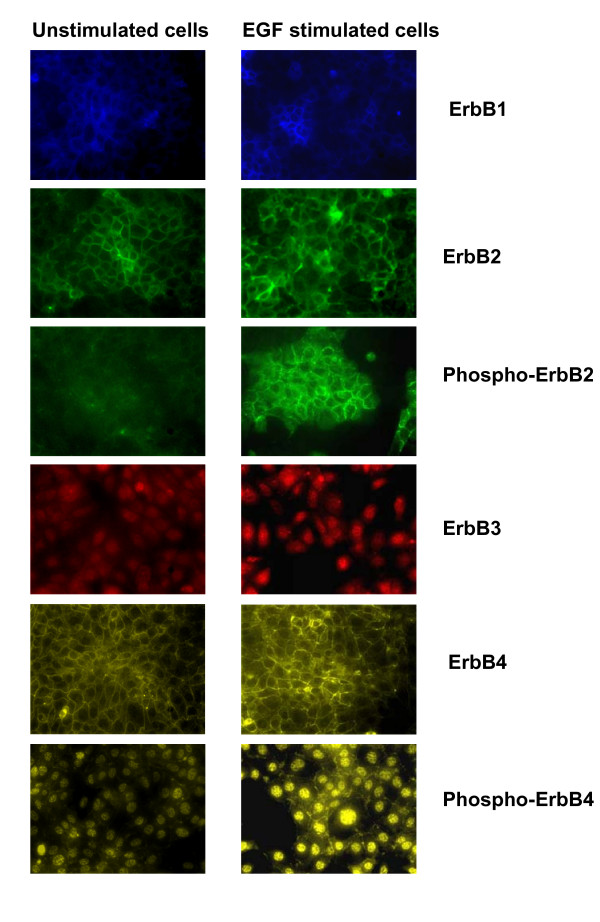
**Immunofluorescent staining of ErbB1-4 receptors, phospho-ErbB2 and phospho-ErbB4 receptors**. +SA mammary tumor cells were initially plated at a density of 2 × 10^5 ^cells/well in 6-well plates (3 replicates/group) and allowed to attach to the plates followed by incubation in mitogen-free media overnight. Afterwards, cells were stimulated with 100 ng/ml EGF for 10 min. The cells were fixed with methanol and incubated with primary antibody directed against specific ErbB receptors, and then incubated with rhodamine conjugated secondary antibody, as described in the Methods section. Magnification is 200×.

Figure 7 shows immunofluorescence images of total ErbB1-4 receptors, phospho-ErbB2 and phospho-ErbB4 receptors, before (Figure 7, left) and 10 min after stimulation with EGF (Figure 7, right). Results showed that the fluorescent intensities of phospho-ErbB2 and phospho-ErbB4 receptors increased after 10 min of EGF exposure. Fluorescent detection of phospho-ErbB1 and phospho-ErbB3 receptors could not be performed because of the unavailability of specific mouse reactive primary antibody for immunofluorescent application. Immunofluorescence studies show that ErbB1, ErbB2, phospho-ErbB2 and ErbB4 receptors are primarily located on the cell surface, ErbB3 receptors are primarily located in the nucleus, while phospho-ErbB4 receptors are relatively evenly distributed between both the nucleus and cell surface in neoplastic +SA mammary epithelial cells (Figure 7).

### Effects of combined γ-tocotrienol treatment with erlotinib or gefitinib on ErbB Receptor Levels and Phosphorylation

**Figure 8:**
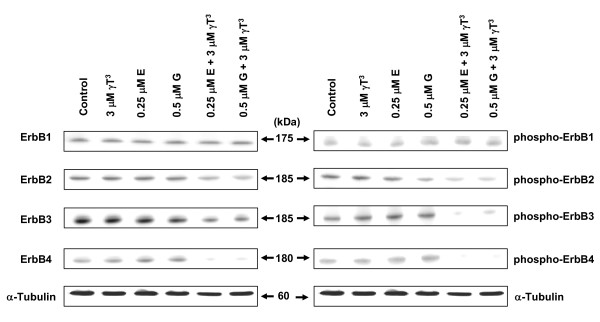
**Western blot analysis of total and phosphorylated ErbB receptor levels treated with γ-tocotrienol (γT^3^), erlotinib (E) or gefitinib (G) either alone or in combination**. +SA mammary tumor cells were plated at a density of 1 × 10^6 ^cells/100-mm culture dish and treated with control or treatment serum-free defined media containing EGF as mitogen. After a 4-day incubation period, whole cell lysates were subjected to PAGE followed by Western blot analysis for total and phosphorylated (active) ErbB receptor levels. Each Western blot is a representative example of data obtained for experiments that were repeated at least three times.

Western blot analysis showed that subeffective doses of erlotinib or gefitinib given in combination with subeffective dose of γ-tocotrienol resulted in a relatively large reduction in EGF-induced ErbB3, ErbB4, and to a lesser extent a decreased in ErbB2 receptor tyrosine phosphorylation as compared to untreated controls (Figure 8, right). Similar treatment was found to have no effect on the level of total ErbB1 or EGF-induced ErbB1 receptor tyrosine phosphorylation (Figure 8, right). Treatment with either γ-tocotrienol, erlotinib or gefitinib alone was not found to cause a change in total or EGF-induced ErbB1-4 receptors tyrosine phosphorylation levels (Figure 8, right). Combination treatment of γ-tocotrienol with erlotinib or gefitinib also caused a reduction in total levels of ErbB2, ErbB3 and ErbB4 receptors, as compared to untreated controls (Figure 8, left).

**Figure 9:**
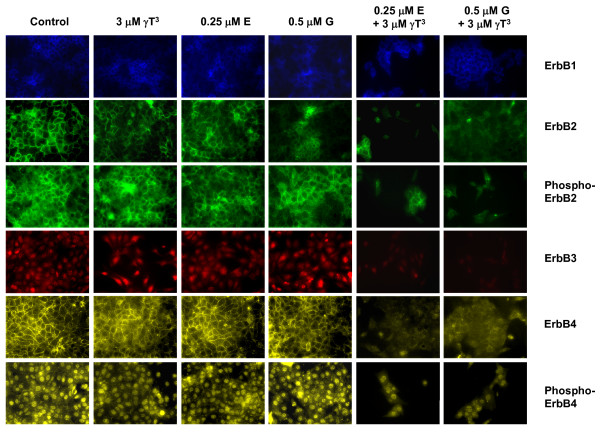
**Immunofluorescent staining of ErbB1-4 receptors, phospho-ErbB2 and phospho-ErbB4 receptors in +SA cells after treatment with, γ-tocotrienol (γT^3^), erlotinib (E), gefitinib (G), alone or in combination**. +SA mammary tumor cells were plated at a density of 2 × 10^5 ^cells per well in 6 well plate (3 replicates/group) and incubated with control or treatment media for 4 days. The cells were then fixed with methanol and incubated with primary antibody directed against specific ErbB receptors followed by rhodamine conjugated secondary antibody, as described in the Methods section. Magnification is 200×.

Figure 9 shows the immunofluorescent images of ErbB receptors after a 4-day treatment exposure. There were fewer cells in all the combination treatment groups, reflecting the growth inhibitory and apoptotic effects of these treatments (Figure 9). The fluorescent images showed that combined treatment of γ-tocotrienol with erlotinib or gefitinib caused a reduction in fluorescence intensities of the total and/or phosphorylated ErbB2-4 receptors, but not ErbB1 receptors. Interestingly, it was found that combined treatment of γ-tocotrienol with erlotinib or gefitinib resulted in a large reduction in fluorescence intensity in the membrane, but only a moderate reduction in the nuclear levels of phospho-ErbB4 in these cells (Figure 9). Fluorescent detection of phospho-ErbB1 and phospho-ErbB3 receptors could not be performed because of the unavailability of specific mouse reactive primary antibody for immunofluorescent application.

## Discussion

Results in this study demonstrate that combined low dose treatment of γ-tocotrienol with erlotinib or gefitinib significantly enhanced the antiproliferative and apoptotic effects of these treatments as compared to monotherapy alone in neoplastic +SA mammary epithelial cells. Studies also showed that combined treatment with low doses of these agents caused suppression in ErbB3, ErbB4, and to a lesser extent, ErbB2 receptor activation in +SA cells. In contrast, trastuzumab treatment had no effect on +SA cell growth when given alone or in combination with γ-tocotrienol.

Vitamin E is a generic term that represents a family of compounds that is further divided into 2 subgroups called tocopherols and tocotrienols. There are 4 natural isoforms exist in each subgroup based on the degree of chromane ring methylation and are classified as α, β, γ and δ tocopherol or tocotrienol [21]. Previous studies have shown that γ-tocotrienol inhibited the growth of +SA mammary epithelial cells at doses that had little or no effect on the normal cell growth [1-3]. Studies have also shown that γ-tocotrienol inhibits the growth of human breast cancer cell lines [29]. Recommended safe dose limits in humans for the various tocotrienol isoforms for human is 200-1000 mg/day and treatment with these doses has been reported to be devoid of adverse side effects [30]. However, very high dose of tocotrienols were reported to inhibit the platelet aggregation and increase clotting time in patients [31]. Treatment doses of γ-tocotrienol used in the present study are physiologically relevant based on serum concentrations of tocotrienols that were found to ranged between 2-4 μM in individuals given a single oral dose of mixed tocotrienols (300 mg) under fed or fasted conditions [32]. The present study shows that treatment with 0.5-3 μM of erlotinib, 1-3 μM gefitinib, or 3.5 μM γ-tocotrienol significantly inhibited EGF-dependent +SA cell growth. Treatment with 0-250 μg/ml trastuzumab alone or in combination with 0-3 μM γ-tocotrienol had no effect on +SA cell growth or viability. The exact reason why trastuzumab was ineffective in suppressing +SA cell growth is presently unknown, but may be related to the fact that not all the breast cancer cells are sensitive to the trastuzumab treatment. Previous investigations have shown that trastuzumab is effective only in cancer cells that are primarily dependent on oncogenic HER/ErbB2 receptor mitogenic signaling [33] whereas other studies using the NIH3T3-HER2 mouse model reported that cancer cells can be ErbB2-dependent, yet still display resistance to trastuzumab treatment [33].

Treatment with 3 μM γ-tocotrienol, 0.25 μM erlotinib, or 0.5 μM gefitinib alone had no effect on +SA cell growth or viability, whereas combined treatment with these agents significantly inhibited +SA cell growth and initiated apoptosis, as evidenced by a large increase in annexin V staining and elevations in cleaved caspase-3 and PARP in these cells. Previous studies have shown that treatment with 10-20 μM γ-tocotrienol was required to initiate apoptosis in +SA cells [2]. Similarly, erlotinib and gefitinib have also been shown to induce apoptosis, but at much greater doses (≥ 1 μM) than those used in the present study [34-36]. Since γ-tocotrienol has been shown to induce caspase-3 cleavage and apoptosis through the activation of caspase-8 (extrinsic pathway) [25], and erlotinib and gefitinib where shown to induce caspase-3 cleavage and apoptosis through the activation of caspase-9 (intrinsic pathway) [35,36], it is possible that apoptosis in the combined treatment groups may be mediated by the activation of both the intrinsic and extrinsic apoptotic pathways. Additional studies are required to determine the exact mechanism initiating apoptosis in the combination treatment groups.

Initial characterization of ErbB receptors in neoplastic +SA mammary epithelial cells showed that ErbB1-4 receptors are expressed in these cells and display tyrosine phosphorylation within 5-10 min following EGF exposure. In addition, total ErbB1-4 receptors levels displayed a gradual decrease between 30-60 min following EGF exposure due to the growth factor-induced receptor endocytosis and degradation. Combined treatment with subeffective doses of γ-tocotrienol and subeffective doses of erlotinib or gefitinib caused a large suppression in ErbB3 and ErbB4 receptor phosphorylation (activation). These findings are of particular interest because elevations in ErbB3 and ErbB4 receptor expression are associated with poor patient prognosis in various types of cancers [37,38]. Previous studies have also shown that ErbB3 receptor heterodimers are more oncogenic and stable, and suppression of ErbB3 receptor tyrosine phosphorylation greatly reduces the tumor cell proliferation and growth [37-39]. These findings suggest that combination therapy with agents that target multiple ErbB receptors effectively interferes with the heterodimer cooperation that exists between the various ErbB receptors.

The exact mechanism responsible for the down regulation in the total levels of ErbB2-4 receptors following combination treatment of γ-tocotrienol with erlotinib or gefitinib is unclear. Recent reports have shown that activated ErbB receptors can form heterodimers with other receptor tyrosine kinases such as IGF receptors, PDGF receptors, and c-Met, and promote breast cancer cell growth and survival [40,41]. Therefore, it is possible that the inhibitory growth effects observed in the combination treatment groups may also involve disrupting ErbB receptor heterodimerization and activation of other types of tyrosine kinase receptors.

Immunofluorescent studies revealed the presence of ErbB1, ErbB2 and ErbB4 receptors on the plasma membrane, whereas ErbB3 receptors were found in the nucleus of neoplastic +SA mammary epithelial cells. These findings are in agreement with previous studies showing ErbB3 receptor localization in the nucleus [42]. The present study also showed intense phospho-ErbB4 receptor staining in the nucleus in addition to plasma membrane even though no ErbB4 staining was observed in the nucleus. These conflicting results can be explained by the proteolytic cleavage of activated ErbB4 receptors and results in the formation of a soluble intracellular tyrosine kinase domain. These liberated intracellular domains will then undergo translocation into the nucleus and function to regulate transcription [43,44]. The phospho-ErbB4 antibody used in the present study detects both the intact and cleaved receptor, and thereby visualizes immunofluorescent staining of this form of the ErbB4 receptor in the membrane and nucleus of +SA cells. In contrast, the antibody used to detect total ErbB4 levels is only able to detect the intact form of the receptor located in the cell membrane and does not detect the cleaved form of the ErbB4 receptor. Nevertheless, these results demonstrate that combined treatment of γ-tocotrienol with erlotinib or gefitinib resulted in a complete inhibition in the phosphorylation of ErbB4 receptors located on the +SA cell membrane, but had little or no effect on nuclear phospho-ErbB4 receptors levels, and strongly suggests that the growth inhibitory effects of combination treatment are mediated through the attenuation of membrane ErbB4 receptor mitogenic signaling, rather than nuclear ErbB4 receptor modulation of transcription.

## Conclusions

Clinical use of agents that target only one member of the ErbB family of receptors have shown limited success in the treatment of cancer. However, results in the present study show that low-dose combination treatment targeting multiple ErbB receptors is more effective in inhibiting mammary tumor cell growth and inducing apoptosis than high dose monotherapy that targets only a single ErbB receptor subtype. These findings strongly suggest that combination therapy may greatly improve therapeutic responsiveness in breast cancer patients.

## Competing interests

The authors declare that they have no personal financial or competing interests. First Tech International Ltd. provided a grant and purified γ-tocotrienol that was used in part to support this research.

## Authors' contributions

SVB carried out experiments, participated in the design of the study, and drafted the manuscript. VBW help with carrying out experiments, and contributed valuable input and discussion regarding the interpretation of the experimental findings. PWS conceived of the study, participated in its design and coordination, and helped to draft the manuscript. All authors read and approved the final manuscript.

## Pre-publication history

The pre-publication history for this paper can be accessed here:

http://www.biomedcentral.com/1471-2407/10/84/prepub
